# Giant congenital melanocytic nevus of the scalp: from clinical-histological to molecular diagnosis

**DOI:** 10.1186/s41065-020-00133-1

**Published:** 2020-05-19

**Authors:** Yi Sun, Yun Zou, Lizhen Wang, Hao Gu, Qingqing Cen, Hui Chen, Xiaoxi Lin, Ren Cai

**Affiliations:** 1 Department of Plastic and Reconstructive Surgery, Shanghai 9th Peoples Hospital Affiliated to Shanghai Jiaotong University School of Medicine, Shanghai, China; 2 Department of oral pathology, Shanghai 9th Peoples Hospital Affiliated to Shanghai Jiaotong University School of Medicine, Shanghai, China; 3 Bio-X Institutes, Key Laboratory for the Genetics of Developmental and Neuropsychiatric Disorders (Ministry of Education), Shanghai Jiao Tong University, Shanghai, P. R. China; 4 Institutes of Biomedical Sciences, Fudan University, Shanghai, P. R. China

## Abstract

Congenital melanocytic nevus (CMN) is a benign proliferative skin disease in the epidermis and dermis. Large to giant CMNs are estimated to be associated with an increased lifetime risk of malignancy. It is necessary to estimate and monitor the risk of malignant transformation for giant CMNs. To date, the clinical “ABCD” criteria and immunohistochemistry studies can be confusing and, to some extent, subjective. Accordingly, the elucidation of genomic analyses of nevi is required to better understand the malignant transformation of CMNs. Here, we describe two large to giant CMNs of the scalp with opposite clinical-histological and molecular evaluations of potential malignancy risk. To our knowledge, this is the first description of a genetic study of large to giant CMNs of the scalp in East Asia. We recommend reviewing the molecular diagnosis together with careful medical history and histological information to facilitate the evaluation of the potential malignancy risk.

## Introduction

Congenital melanocytic nevus (CMN) is defined as a benign proliferative skin disease in the epidermis and dermis. It is usually apparent at birth and progressively grows with individuals, with an incidence rate in newborns of approximately 1–2% and no sexual bias [1, 2]. CMNs can be characterized as a papular, rugose, pebbly, verrucous, or even cerebriform surface and may even exhibit darker and thicker pigmented hairs [2]. It is well known that the major concern of CMNs is the risk of malignant transformation. Large to giant CMNs are estimated to be associated with an increased lifetime risk of melanoma of 3 to 11% [2, 3]. Nevertheless, the exact magnitude of the risk is still unknown [4, 5]. In general, the treatment options, including full or partial excision, curettage, laser treatment or a combination of these methods [6, 7], aims to reduce the risk of malignancy [8, 9]. However, there is no consensus on the most appropriate strategy for patients with giant CMNs because these lesions vary in size and location and may involve vital structures or deep anatomic zones; thus, partial or complete removal is difficult. In addition, nevi cells may be left behind after excision because of the deep extension of nevi cells along skin appendages, even into skeletal muscle [10]. In this case, it is necessary to estimate and monitor the risk of malignant transformation for giant CMNs.

The clinical “ABCD” criteria (asymmetry, border, color, and dimension) introduced for the visual recognition of early melanoma hold practical value in current clinical settings. However, this evaluation of a pigmented lesion is largely subjective [11, 12]. Moreover, immunohistochemistry studies are still controversial because of the variability and discordance in diagnostic criteria [13, 14]. A study in 1996 assessed the interobserver agreement on the diagnosis of cutaneous pigmented lesions within four experienced histopathologists and found considerable disagreement among the pathologists on the diagnosis of benign pigmented lesions versus melanoma [15]. In addition, the elucidation of genomic analyses of nevi is required to better understand malignant transformation. According to the literature, CMN frequently harbors activating NRAS or BRAF (V600E) mutations. There is no evidence explaining the increased malignant transformation of CMNs with mutations in NRAS [16]. However, the BRAF (V600E) mutation, the predominant oncogene associated with melanoma, may explain the transition from benign neoplasm to malignancy [17].

In this case series, we report two patients with large to giant CMNs of the scalp. Interestingly, one patient who we diagnosed with low-risk CMN harbored a BRAF (V600E) somatic mutation. In contrast, neither BRAF (V600E) nor NRAS (Q61R/L) was detected in the other patient we diagnosed as relatively high risk, suggesting that molecular diagnosis should not be neglected. To our knowledge, this is the first report of a genetic study of large to giant CMNs of the scalp in East Asia.

## Methods

This study was conducted following the principles outlined in the Declaration of Helsinki and was authorized and approved by the Ethics Committee of Shanghai Ninth People’s Hospital Affiliated to Shanghai Jiaotong University School of Medicine (equivalent to the Institutional Review Board). Verbal and written informed consent for study participation and publication of identifying information and images was granted for each child’s patients prior to the study. All patients were assessed by at least 2 plastic surgeons and found to fit the criteria for CMN according to the consensus classification and standardized categorization of the cutaneous features of CMN as previous published [18]. Clinical data, including a careful medical history provided by the patients, physical examination, histopathological findings and genetic analysis results, were recorded.

**Fig. 1:**
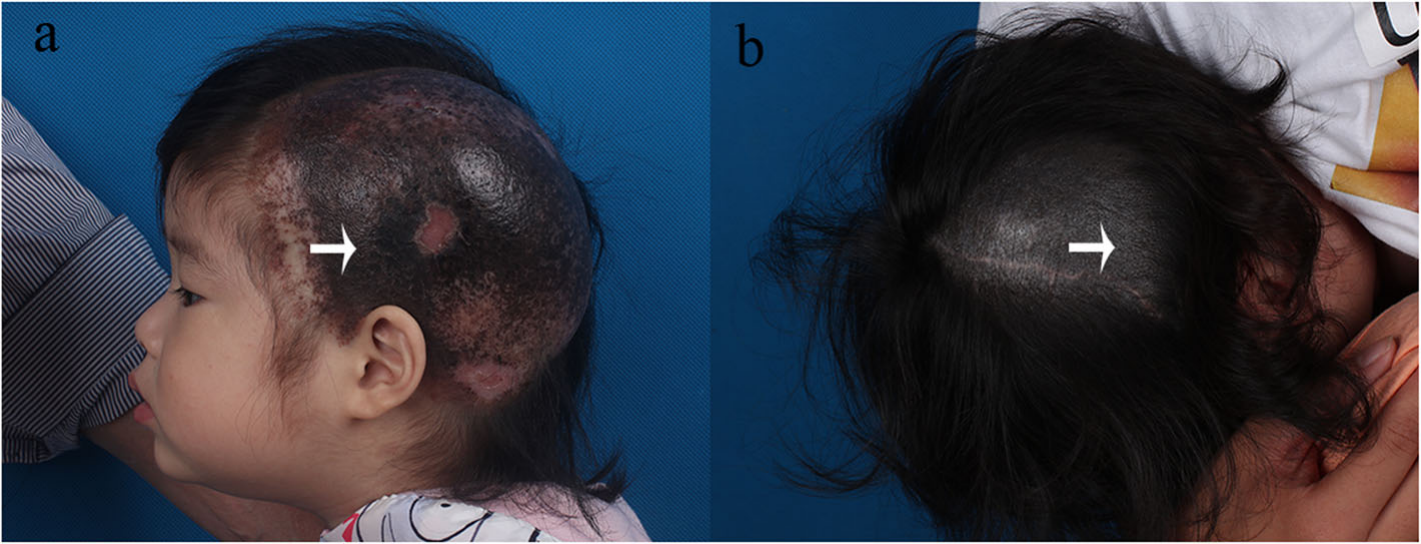
**a** Clinical manifestation of the patient#1: left-sided giant congenital melanocytic nevi of the scalp. The lesion was flat and asymmetric with irregular boundary and uneven pigmentation. **b** Clinical manifestation of the patient#2: large congenital melanocytic nevi on the right side of the scalp. The lesion was flat and symmetric with uniform pigmentation

Patient #1, a 2-year-old girl, presented at our clinic with left-sided giant congenital melanocytic nevi of the scalp (Fig. 1a). The patient was born with black lesions on her left scalp, and the lesions progressively became larger with age. The lesion was flat and asymmetric with irregular boundary and uneven pigmentation. A medical history of repetitive erosions and ulcerations for 2 years was mentioned. Patient #2 was a 10-month-old girl with a large congenital melanocytic nevus on the right side of the scalp (Fig. 1b) with an unremarkable medical history. The nevus was darkly pigmented at birth, and there were no satellite nevi. The lesion was flat and symmetric with uniform pigmentation. During the physical examination, we discovered no hair on the nevi lesions in either patient. From the family history, both nevi lesions became darker with age, and spontaneous regression was not observed since birth. In addition, no prenatal complications were mentioned in either patient. A clinical evaluation was completed by two experienced plastic surgeons based on the “ABCD” clinical criteria.

To determine the histological and genetic changes, we performed minimally invasive biopsy (D = 2 mm, all layer biopsy) under local anesthesia (Penles and 1% xylocaine) on the nevi lesions of the scalp (Fig. 1, arrows). In addition to tissue specimens, blood specimens from the peripheral vein were also collected. One tissue specimen was sent to our institution’s Department of Pathology for histopathological analysis and was fixed in 10% formaldehyde solution, embedded in paraffin, sectioned, and stained with hematoxylin-eosin. Pathology analysis was performed by two qualified and experienced pathologists. The other tissue specimens were preserved in liquid nitrogen for DNA extraction and NGS.

We extracted genomic DNA from peripheral blood using the QIAamp DNA Blood Mini Kit (QIAGEN, Germany) according to the manufacturer’s instructions. Minimally invasive biopsy was performed on the nevi lesions via a standard procedure. DNA was extracted from the biopsy tissues by using the QIAamp DNA Mini Kit (QIAGEN, Germany) with overnight incubation in proteinase K at 56 °C for approximately 14 h. A Qubit 2.0 fluorimeter (Life Technologies, Carlsbad, CA, USA) and a Thermo NanoDrop 2000 spectrophotometer (Thermo, Wilmington, DE, USA) were used to determine the DNA concentration and quality.

The targeted gene panel of NGS was designed with the most common genetic alterations of CMN in the RAS-RAF-MEK signaling pathway, including RAS family (NRAS, KRAS, HRAS) and BRAF, as well as MAP2K1, the downstream signal in the RAS-RAF-MEK pathway [19, 20]. The library construction was performed by the recommendations of Illumina (San Diego, CA, USA). DNA was sheared, purified, end-repaired, adenylated on the 3′ ends, ligated with Illumina adaptors and amplified by PCR. A series of probes were designed and synthesized by the IDT (Integrated DNA Technologies) company (Coralville, USA) to target the exons and exon/intron boundaries of the genes. After target capture and purification, the quantity of the library was validated using quantitative PCR (Kapa Biosystems, Wilmington, MA), and the integrity was validated using a 2100 Bioanalyzer (Agilent Technologies, Santa Clara, USA). The libraries were sequenced by HiSeq-series sequencing systems (Illumina, San Diego, USA). The bioinformatics pipeline, including GATK (version 3.4), BWA (version 0.7.13) and VarDict, was used to analyze the mutation status of NRAS, KRAS, HRAS, MAP2K1 and BRAF. The results were also manually confirmed using Integrative Genomics Viewer (IGV). Single nucleotide variants with a variant allele frequency of 1% or greater were ultimately selected.

## Results

According to the clinical diagnostic criteria of “ABCD” and a history of erosion ulcerations outside of the neonatal period, patient #1 was diagnosed with giant CMN with a high risk of malignant transformation, while patient #2 was determined to have a relatively lower risk of malignancy.

**Fig. 2:**
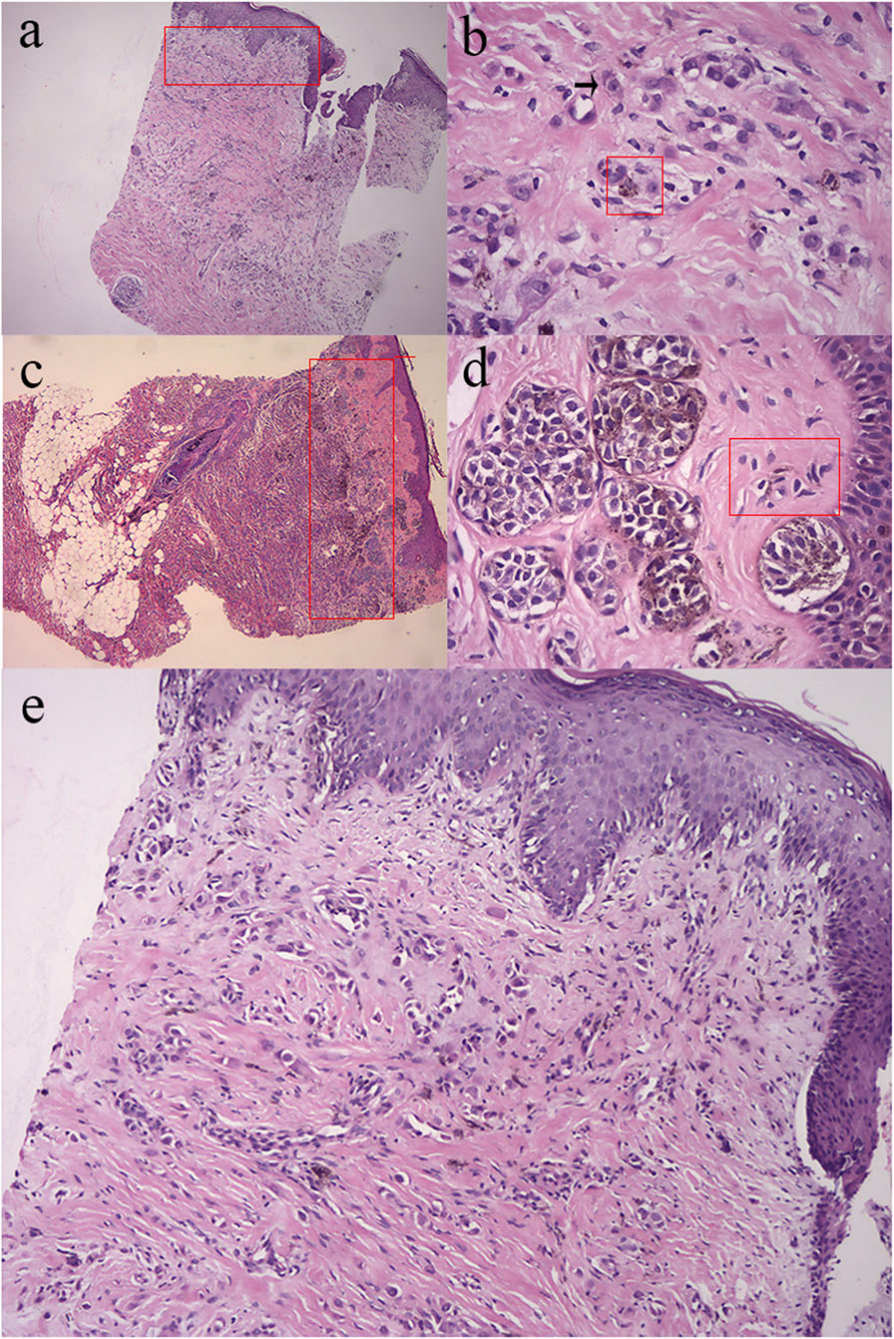
**a** Predominantly nested intraepidermal melanocytes (rectangle) in patient#1 (hematoxylin–eosin, original magnification × 40). **b** Predominantly as single cells (rectangle) in patient#2 (hematoxylin–eosin, original magnification × 40). **c** Predominantly small pigment granules (rectangle) and obvious pleomorphism of nucleolus and cytoplasmic abundance (arrow) in patient#1 (hematoxylin–eosin, original magnification × 400). **d** Predominantly thick pigment granules (rectangle) and no obvious cytologic atypia in patient#2 (hematoxylin–eosin, original magnification × 400). **e** Epidermal thickness and fiber proliferation in patient#1 (hematoxylin–eosin, original magnification × 100)

Significant differences were found in the histomorphological features between the two patients. These differences included nesting of intraepidermal melanocytes (predominantly nested intraepidermal melanocytes in patient #1 vs predominantly as single cells in patient #2, Fig. 2a,b), pigment granules (small granules in patient #1 vs thick granules in patient #2, Fig. 2c,d) and cytologic atypia (obvious pleomorphism of nucleolus and cytoplasmic abundance in patient #1 vs no obvious cytologic atypia in patient #2, Fig. 2c,d). Additionally, epidermal thickness and fiber proliferation were more frequently observed in patient #1 (Fig. 2e).

**Fig. 3:**
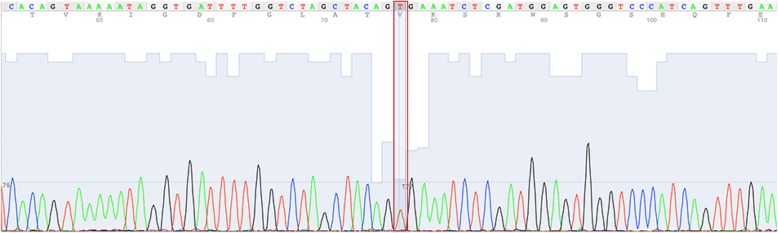
Sanger sequence of somatic BRAF(7:140453136 A > T)

Molecular analysis of the DNA extracted from the skin lesion confirmed a somatic mutation in the BRAF gene (V600E) at a mutation frequency of 29.7% in patient #2 (Fig. 3). NGS analysis showed a depth of coverage for this mutation of 5567x. In contrast, for mutations in the MAPK signaling pathway, neither BRAF (V600E) nor NRAS (Q61R/L), were detectable in patient #1.

## Discussion

In this study, we report two patients with large to giant CMNs of the scalp, in which genetic diagnosis added more information in risk evaluation to a single clinical-histological study. Patient #1 was diagnosed with a high risk of malignant transformation based on the clinical manifestations according to the “ABCD” criteria, while patient #2 was diagnosed with a low risk. Findings of histomorphological features such as nesting of intraepidermal melanocytes, pigment granules and cytologic atypia confirmed this suspicion at the histological level. In contrast, molecular diagnosis led to the opposite conclusion: the malignancy rate in patient #2 is relatively higher than that in patient #1 because of the detected BRAF (V600E) mutation. The mechanism of malignant transformation in CMNs has not been well elucidated. According to the literature, melanoma frequently harbors the BRAF (V600E) mutation [21]. BRAF (V600E) was also identified in CMNs [22], suggesting that the alteration constituted an early key somatic event in malignant transformation.

The spontaneous regression of large to giant CMNs in natural history has been reported many times since 1988 [23–25]. Kageshita T et al. [24] reported the spontaneous evolution of CMNs and marked cosmetic improvement of five scalp CMNs over the first 2 years of life. Margileth AM et al. [25] reviewed 17 children with scalp CMNs and found spontaneous regression occurring in 6 patients. In this study, we did not report spontaneous regression in either patient, which seemed to be related to ethnic differences. As noted by Polubothu and Kinsler [26], the final color of the nevus is related to the genetically determined skin color and inherited pigmentary phenotype of the individual and not the nevus color in the first 3 months of life. Children with a lighter normal skin color will have more lightening of the CMN. Despite the clinical disappearance of CMNs, the potential risk of malignant transformation cannot be completely eliminated. Vilarrasa E et al. [27] found persistence of the nevi cells deep in the dermis, even extending to the subcutaneous tissues in two children with large to giant scalp CMNs and spontaneous involution. Negligence of the evaluation of malignant changes may have important consequences.

Genetic studies add information for the diagnosis and evaluation of malignant potential, and the subsequent molecular targeted drugs also provide new management strategies for giant CMNs since traditional treatment relies heavily upon iterative surgical procedures, such as excision and curettage. Mir A et al. [28] reported the first *BRAF*-mutated giant CMN treated with trametinib, an MEK inhibitor. The color and extent of the nevus lesion improved, and the patient’s intractable pain and pruritus rapidly resolved. By using a xenograft model, Rouille et al. [29] reported that the local administration of MEK and Akt inhibitors can limit the proliferative potential of CMNs, supporting the continued investigation of targeted therapies in large to giant CMNs.

This study emphasizes the limitation of a single clinical-histological diagnosis and the importance of NGS. The tendency of malignant transformation in large to giant CMNs should never be neglected. We recommend reviewing the molecular diagnosis together with careful medical history and histological information to facilitate the evaluation of the malignant potential risk. Patients, especially those with large to giant CMNs, should receive careful physical examination, medical history taking, histopathological analysis and genetic testing. Physicians should be aware of the potential risk of malignant transformation. Patients should have regular clinical follow-ups or obtain appropriate initial treatment, such as molecular-targeted drugs.

## Conclusion

In this case series, we reported two large to giant CMNs with opposite clinical-histological and molecular evaluations of malignant potential risk. To our knowledge, this is the first description of a genetic study of large to giant CMNs of the scalp in East Asia. It would be valuable to obtain relevant molecular information with a clinical and histological diagnosis. Such information would help us understand and evaluate the potential risk of malignancy in patients with large to giant CMNs.

## Data Availability

All data used during the study appear in the submitted article.

## Abbreviation

CMN: Congenital melanocytic nevus
